# Severe Fatigue Is Common Among Pediatric Patients with Primary Immunodeficiency and Is Not Related to Disease Activity

**DOI:** 10.1007/s10875-021-01013-7

**Published:** 2021-03-17

**Authors:** Linde N. Nijhof, Marco van Brussel, Esther M. Pots, Raphaële R. L. van Litsenburg, Elise M. van de Putte, Joris M. van Montfrans, Sanne L. Nijhof

**Affiliations:** 1 Department of Pediatrics, Wilhelmina Children’s Hospital, University Medical Center Utrecht, Surface mail: HP KE.04.133.1, Post box 85090, 3508 AB Utrecht, The Netherlands; 2 Department of medical physiology, Child Development and Exercise Center, Wilhelmina Children’s Hospital, University Medical Center Utrecht, Utrecht, The Netherlands; 3 Princess Máxima Center for Pediatric Oncology, Utrecht, The Netherlands; 4 Department of Pediatric Immunology and Infectious Diseases, Wilhelmina Children’s Hospital, University Medical Center Utrecht, Utrecht, The Netherlands

**Keywords:** Pediatric, fatigue, immunodeficiency, disease activity, health-related quality of life

## Abstract

**Purpose:**

Fatigue is a distressing symptom commonly reported among pediatric patients with primary immunodeficiency (PID). However, the relationship between fatigue and disease activity is currently unknown.

**Methods:**

In this cross-sectional study, we examined the prevalence of severe fatigue, the effect of fatigue on health-related quality of life (HRQoL), and the effects of disease activity and comorbidity on fatigue severity among pediatric patients 2–18 years of age with PID. Fatigue and HRQoL were assessed using the pediatric quality of life inventory multidimensional fatigue scale (PedsQL MFS) and generic core scales (PedsQL GCS), respectively. Linear regression analyses and an analysis of covariance were used to compare the fatigue scores with the scores obtained from a healthy control group. Data were adjusted for age and sex.

**Results:**

Of the 91 eligible patients, 79 were assessed (87% participation rate), with a mean age of 10.4 ± 4.4 years. Pediatric patients with PID reported significantly higher fatigue levels compared to healthy peers, with an 18.9% prevalence of severe fatigue. Moreover, higher fatigue levels were inversely associated with HRQoL in all domains and directly associated with school absences. We found that severe fatigue was comparable between common variable immunodeficiency (CVID), combined immunodeficiency (CID), and selective immunoglobulin A deficiency (SIgAD) patients, but was not reported in the X-linked agammaglobulinemia (XLA) patients studied. Finally, fatigue severity was not significantly associated with disease activity or comorbidity.

**Conclusions:**

Nearly 20% of pediatric patients with PID reported experiencing severe fatigue, and fatigue was reported among a wide range of PID subcategories. In addition, severe fatigue negatively affected the patient’s quality of life and daily functioning, but was not associated with disease activity or comorbidity. Thus, targeting severe fatigue might be a promising strategy for improving the overall well-being and quality of life of pediatric patients with PID.

## Introduction

Primary immunodeficiency (PID) is a heterogeneous group of rare disorders characterized by reduced or absent function of the innate and adaptive immune systems, resulting in increased susceptibility to infection and autoimmune disease [1, 2]. The estimated global prevalence of PID is 29.1–50.5 individuals per 100,000 people [3]. Major advances in pediatric medicine have resulted in many patients with PID transitioning from having a potentially fatal disease to living with a chronic condition [4]; however, this has led to a new set of challenges, as growing up with a chronic disease can place considerable psychological, social, emotional, and financial burden on the children themselves, as well as their relatives [5, 6]. Indeed, chronically ill children frequently report having lower health-related quality of life (HRQoL) compared to their healthy peers [7, 8]. In addition to reduced HRQoL, children with PID also have an increased risk of psychological problems and experience limitations in both physical and social activities [9–14].

Although fatigue is a relatively common symptom among children with PID, negatively affecting their well-being and participation in daily life, the precise prevalence of severe fatigue and possible associated factors are currently unknown [8, 11, 15]. Using retrospective data collected from the US Immunodeficiency Network (USIDNET) patient registry, Hajjar et al. recently estimated that the prevalence of fatigue among adults with PID is 18%, compared to 6–7.5% in the general population [4]. Interestingly, recent research reported a similar prevalence of fatigue among children with a chronic condition (21.1%), regardless of the type of underlying condition [8]. It appears that fatigue severity can be better explained by transdiagnostic psychosocial factors—for example, coping strategies, cognitive, emotional, and motivational factors underlying neurobiological factors and universal protective factors such as resilience—rather than specific characteristics of the disease [8].

Most studies that evaluate fatigue among children and adolescents have been limited to describing the intensity and/or duration of fatigue. Consequently, the effects of fatigue on daily living, as well as the relationship between disease activity and fatigue, have been studied only rarely in patients with PID.

The wide-ranging effects of fatigue illustrate the relevance and urgency of studying and designing interventions for pediatric patients with PID. Importantly, early detection and timely intervention may improve the patients’ well-being and their participation in daily life, possibly preventing the persistence of chronic fatigue into adulthood, improving long-term quality of life. Here, we studied (1) the prevalence of severe fatigue among children and adolescents with different subtypes of PID using self-reported and parent-proxy–reported questionnaires, comparing the results with healthy peers; (2) the effect of fatigue on health-related quality of life (HRQoL); and (3) the putative association between fatigue and various factors such as disease activity and comorbidity. Finally, we discuss the consequences of these findings with respect to potential interventions.

## Materials and Methods

### Study Design and Study Population

In this cross-sectional, single-center study, we recruited pediatric patients (2–18 years of age) with PID who visited the pediatric Immunology and Infectious Diseases outpatient clinic at the Wilhelmina Children’s Hospital, part of the University Medical Center in Utrecht, the Netherlands, from February 2017 through April 2017. Patients who were diagnosed with PID in accordance with the European Society for Immunodeficiencies (ESID) criteria were eligible [16]. Patients whose participation would have caused them to be involved in more than one research project were excluded in order to minimize study burden and the risk of study interference. This study was conducted in accordance with the Declaration of Helsinki and was part of a national observational multicenter study in primary immunodeficiency patients. The study was approved by the Medical Research Ethics Committee of the Erasmus Medical Center Rotterdam (protocol number 13-700). Local ethical approval at the Medical Research Ethics Committee of University Medical Center Utrecht was obtained for this sub-study. All participants and their caregivers/parents provided written informed consent before participating.

### Data Collection

Both verbal and written information regarding the study were provided during inpatient hospitalization and outpatient clinical visits or by phone and regular mail sent 2 weeks before the patient’s visit to the outpatient clinic. General patient characteristics such as age, sex, ethnic background, disease duration, and comorbidity were extracted from the medical records and are summarized in Table 1. Patients and/or their parents received hard-copy questionnaires through regular mail.

**Table 1:** Demographic characteristics and disease activity of the study groups

	Patients	Healthy peers	Difference in mean values (95% CI)	*p* value
	*n* = 79	*n* = 504		
Sex, male (%)	59.5	47.6		**0.05**^a^
Age at participation, years, mean ± SD	10.4 ± 4.4	9.5 ± 4.7	−0.88 (−2.0; 0.2)	0.33^b^
Ethnic background n (%)				
Northern European (Caucasian)	75 (94.9%)			
Turkish	2 (2.5%)			
Asian Surinamese	1 (1.3%)			
Southern European	1 (1.3%)			
Disease duration, months, mean (range)	64.8 (1–156)			
Diagnostic category, *n* (%)				
CVID	24 (30.4%)			
SIgAD	20 (25.3%)			
CID	6 (7.6%)			
XLA	5 (6.3%)			
Other^c^	24 (30.4)			
Comorbidity category (%)^d^	*n* = 16^e^			
Atopic	6.3%			
Autoimmune	8.9%			
Syndrome	2.5%			
Neurological	3.8%			
Congenital	5.1%			
Psychiatric	7.6%			
Disease activity (%)^f^	*n* = 79			
Low	72.2%			
Moderate	26.6%			
High	1.3%			

Significant results *p* < .05 are presented in bold

Abbreviations: *CI*, confidence interval; *CVID*, common variable immunodeficiency; *SIgAD*, selective immunoglobulin A deficiency; *CID*, combined immunodeficiency; *XLA*, X-linked agammaglobulinemic; *SD*, standard deviation

^a^Chi-square test

^b^Independent sample student’s *t* test

^c^Other includes specific antibody deficiency, WHIM (warts, hypogammaglobulinemia, infections, and myelokathexis) syndrome, and adenosine deaminase 2 (DADA2) deficiency

^d^Comorbidities were categorized as follows: atopic (asthma, eczema, allergies), autoimmunological (inflammatory bowel disease, celiac disease), syndromic (22Q11 deletion, Turner syndrome), neurological (epilepsy, migraine, developmental disorder), congenital (congenital heart disease, growth hormone deficiency, congenital foot defects), and psychiatric (attentional (hyperactivity) deficit disorder, autism, depression, and anxiety disorder)

^e^Two patients had one comorbidity, and 14 patients had two or more comorbidities

^f^Based on the disease activity score (see Methods for details)

### Measurement Instruments and Psychometric Characteristics

Two validated questionnaires used to assess fatigue and HRQoL were completed at home. All measurement instruments were completed as either parent-proxy–reported (for children 2–7 years of age, completed by one parent) or self-reported (for children and adolescents 8–18 years of age); in addition, one parent of each child 8–18 years of age also completed a parent-proxy report. Prior research reported that in general, paternal and maternal proxy HRQoL ratings of their child are interchangeable [17]. Parents and children were instructed to complete their questionnaires separately, as the presence of the parents can influence the child’s response, and previous studies have shown that self-reported and proxy-reported questionnaires are not necessarily interchangeable [18–20]. Specific data were recorded as missing when a parent and/or child omitted one or more items on the questionnaire(s).

The primary outcome in this study was fatigue measured using the pediatric quality of life inventory multidimensional fatigue scale 3.0 (PedsQL MFS), which is recommended for studying both self-reported fatigue and parent-proxy–reported fatigue in pediatric patients with a chronic illness [18]. The PedsQL MFS consists of 23 items (total fatigue) with three subscales measuring general fatigue, sleep/rest fatigue, and cognitive fatigue, yielding a score for each subscale ranging from 0 to 100 points; lower scores indicate more fatigue experienced in the previous month. The general fatigue scale contains questions regarding the subjective feeling of fatigue and the energy required to execute activities; the sleep/rest fatigue scale contains questions regarding the quantity and quality of sleep and rest; finally, the cognitive fatigue scale contains questions regarding attention and memory. The Dutch version of the PedsQL was reported to have good internal consistency, with Cronbach’s alpha >0.70 [21]. To quantify the number of severely fatigued children and adolescents, we used a cutoff value of the general fatigue subscale set at 2 standard deviations (2SD) below the mean, taking into account both sex and age (2–4, 5–7, 8–11, and 12–18 years). The data were then compared with a reference population collected from October 2009 through May 2010 at daycare facilities and schools in the Netherlands [22].

Secondary outcomes included disease activity and comorbidity. Because no standardized measurement is currently available for determining disease activity in PID, the level of infectious complications and/or inflammatory disease for each patient was obtained in consultation with a pediatric immunologist or clinical expert in the field and was measured in a 3-month window around the time in which the questionnaires were completed. The level of disease activity was classified as low, moderate, or high based on the following criteria: “low” refers to no extra medication in addition to usual care (immunoglobulins/antibiotics); “moderate” refers to the patient taking one additional medication to treat his/her PID, in addition to usual care; and “high” refers to the patient requiring a significant amount of additional outpatient visits, daycare visits, hospitalizations, or immunosuppressive drugs compared to the standard treatment and follow-up scheme. The scoring was performed by two independent observers (LN and EP), and incongruences were resolved by the third author (JM).

We also recorded comorbidity for each patient. When present, we classified each comorbidity into one of the following categories: atopic, autoimmune, syndrome, neurological, congenital, or psychiatric.

School absences were measured retrospectively as the percentage of nonappearance at school in 2 weeks and 6 months prior to completing the questionnaire. Self-reported percentages were confirmed with the parents and were found to be identical (data not shown).

In addition, we used the PedsQL generic core scales (PedsQL GCS) to assess total HRQoL in the past month [23]. The PedsQL-GCS is a questionnaire that is widely used in many different populations with chronic diseases [10, 17, 24, 25]. This makes the comparison with other countries and disease groups possible. Moreover, this questionnaire has a parent-proxy and self-reported version, thus providing a good overview of the development of HRQoL across different ages. The PedsQL GCS consists of the following four subscales (each with a score ranging from 0 to 100): physical functioning, emotional functioning, social functioning, and functioning at school. Higher scores indicate higher HRQoL and better functioning. This instrument has good validity and internal consistency reliability, with Cronbach’s coefficient alpha >0.70) [21, 23].

### Data Analysis

Descriptive statistics were used to summarize the clinical characteristics and the prevalence of severe fatigue (defined as 2SD below the norm of healthy peers, which was used as a reference group). The baseline characteristics of the patients and the reference group were compared using an independent-sample student’s *t* test or Pearson’s chi-square test, as appropriate.

Normally distributed data from the primary outcome measures are presented as mean ± SD; except where indicated otherwise, all other data are presented as median values.

The fatigue scores of the children and adolescents with PID were compared with the reference group using linear regression and are reported as the mean difference with a 95% confidence interval (CI). Separate analyses were performed for parent-proxy–reported data for children 2–7 years of age and children/adolescents 8–18 years of age.

Both age and sex have been reported as important determinants of fatigue, including chronic fatigue [21, 26]. Therefore, these two variables were used as covariates in all analyses.

The relationship between general fatigue and HRQoL was analyzed using linear regression. The relationships between fatigue and disease activity and between fatigue and comorbidity were analyzed using the beta coefficient (B) with 95% CI based on multivariable linear regression, with outcome (general fatigue) as the dependent variable and disease activity (low or moderate) and comorbidity (yes or no) as independent variables. Only one patient scored high for disease activity and was therefore included in the regression analysis as having moderate disease activity. The same model was used when adjusting for covariables.

Any missing values in the PedsQL GCS and PedsQL MFS were handled in accordance with the scoring guidelines. Specifically, missing scores were calculated by imputing the mean value of the completed items in that scale, provided at least 50% of the items in that scale were completed [27].

The significance level for all group comparisons and regression modeling was set at *p* < 0.05. All analyses were performed using SPSS Statistics for Windows, version 20.0 (IBM Corp., Armonk, NY).

### Sample Size Calculation

The sample size was calculated using G*Power version 3.1.9.2 [28]. Type 1 error was set at 0.05, and type 2 error was set at a power of 80%. A medium effect size (0.15) was used. Thus, a sample size of 77 patients with PID was needed in order to provide sufficient power for this study.

## Results

### Study Population

A total of 92 children and adolescent patients, ranging from 2 to 18 years of age, were invited to participate in the study. One patient was subsequently excluded due to concurrent involvement in other research projects. A total of 79 of the 91 parent-proxy–reported questionnaires (86.8%) and 52 of the 55 self-reported questionnaires (94.5%) were completed and were used to assess fatigue and HRQoL in these children. Five diagnostic categories were distinguished in the patient cohort (Table 1). Of the 79 participating children, 16 (20.3%) had comorbidity, two patients (2.5% of the entire cohort) had one comorbid diagnosis, and 14 patients (17.7%) had two or more comorbid diagnoses. The demographics and other characteristics of the patient and control groups are summarized in Table 1. As part of routine outpatient clinical care, all CVID, CID, and XLA patients were treated with immunoglobulin replacement therapy (either i.v. or s.c.). Patients with recurrent infections despite immunoglobulin replacement therapy or with bronchiectasis received antibiotic prophylaxis with co-trimoxazole in addition (24 mg/kg/day). The SIgA-deficient patients were all symptomatic (i.e., suffering from recurrent airway infections) and received antibiotic prophylaxis with co-trimoxazole during periods of the year with high incidences of respiratory infections. In the case of autoimmune pathology, patients were treated with the designated treatment protocols for each specific autoimmune disease. Deviations from this standard scheme resulted in a higher disease activity score.

### Comparison of Self-Reported Fatigue Between Pediatric Patients with PID and Healthy Peers

Among the pediatric patients with PID, 18.9% were scored as severely fatigued (defined as >2SD below the norm of healthy peers), compared to 4.4% of the healthy peer group (*p* < 0.005).

Compared to the patient group as a whole, severe fatigue was reported in CVID, CID, and SIgA-deficient patients alike, but was not reported in XLA patients. Nevertheless, it is important to note that this study was not powered to compare levels of severe fatigue between the different categories of immunodeficiencies; thus, these findings are descriptive at best.

Moreover, as shown in Table 2, the patients 8–18 years of age self-reported significantly more fatigue than their peers, controlled for age and sex with respect to total fatigue, general fatigue, and sleep/rest fatigue, but scored similarly with respect to cognitive fatigue.

**Table 2:** Fatigue scores for the two study groups

	Patients	Healthy peers	Difference in mean values (95% CI)	*p* value
PedsQL MFS (range: 0–100)^d^—*self-reported* *8–18 years of age*	*n* = 52	*n* = 366		
Total fatigue	69.7 ± 16.9	76.8 ± 12.7	−6.7 (−10.7; −2.8)^c^	**<0.005**^**b**^
General fatigue	68.9 ± 19.8	80.3 ± 14.4	−10.2 (−14.6; −5.8)^c^	**<0.005**^**b**^
Sleep/rest fatigue	68.6 ± 20.6	74.5 ± 15.6	−5.2 (−10.0; −0.4)^c^	**<0.005**^**b**^
Cognitive fatigue	71.6 ± 20.1	75.7 ± 18.2	−4.8 (−10.3; 0.7)^c^	.09^b^
Percentage severely fatigued PID—total group, *n* (%)	53 (18.9%)	366 (4.4%)		**<0.005**^**a**^
CVID	21 (28.6%)			
SIgAD	4 (25.0%)			
CID	5 (20.0%)			
XLA	5 (0.0%)			
Other^e^	18 (11.1%)			
PedsQL MFS (range: 0–100)^d^—*parent-proxy–reported* *2–7 years of age*	*n* = 24	*n* = 187		
Total fatigue	61.8 ± 16.6	82.9 ± 10.9	−21.1 (−26.1; −16.1)^c^	**<0.005**^**b**^
General fatigue	52.4 ± 21.0	83.5 ± 12.1	−31.1 (−36.8; −25.4)^c^	**<0.005**^**b**^
Sleep/rest fatigue	62.7 ± 18.7	85.1 ± 12.9	−22.4 (−28.2; −16.5)^c^	**0.011**^**b**^
Cognitive fatigue	70.8 ± 18.1	80.1 ± 16.3	−9.3 (−16.4; −2.3)^c^	.306^b^
Percentage severely fatigued—total group, *n* (%)	24 (62.5%)	187 (2.7%)		**<0.005**^**a**^
CVID	2 (50.0%)			
SIgAD	15 (80.0%)			
CID	1 (0.0%)			
XLA	0 (N.A.)			
Other^e^	6 (33.3%)			
PedsQL MFS (range: 0–100)^d^—*parent-proxy–reported* *8–18 years of age*	*n* = 54	*n* = 310		
Total fatigue	66.2 ± 20.4	80.2 ± 13.4	−14.0 (−18.2; −9.8)^c^	**<0.005**^**b**^
General fatigue	61.0 ± 24.2	80.0 ± 15.2	−18.9 (−23.8; −14.1)^c^	**<0.005**^**b**^
Sleep/rest fatigue	68.8 ± 21.1	83.1 ± 13.9	−14.3 (−18.8; −9.8)^c^	**<0.005**^**b**^
Cognitive fatigue	69.4 ± 23.1	77.5 ± 18.8	−8.1 (−13.7; −2.4)^c^	**0.005**^**b**^
Percentage severely fatigued—total group, *n* (%)	54 (38.9%)	310 (2.9%)		**<0.005**^**a**^
CVID	21 (61.9%)			
SIgAD	5 (60.0%)			
CID	5 (40.0%)			
XLA	5 (0.0%)			
Other^e^	18 (16.7%)			

Significant results *p* < .05 are presented in bold

Abbreviation: *N.A.*, not applicable

Data are presented as the mean ± SD, except where otherwise indicated

^a^Chi-square test

^b^Linear regression model

^c^Adjusted for age and sex

^d^The PedsQL multidimensional fatigue scale is scored on a scale from 0 to 100, with lower scores indicating more fatigue. A negative difference indicates a lower mean score for the children with PID, indicating more fatigue

^e^Other includes specific antibody deficiency, WHIM (warts, hypogammaglobulinemia, infections, and myelokathexis) syndrome, and adenosine deaminase 2 (DADA2) deficiency

### Comparison of Parent-Proxy–Reported Fatigue Between Patients with PID and Healthy Peers

The parent-proxy–reported fatigue scores of patients with PID and the fatigue scores reported by the parents of healthy children 2–7 years of age and 8–18 years of age are summarized in Table 2. Compared to the healthy peer group of children 2–7 years of age, the parents of children 2–8 years of age with PID reported significantly more fatigue for their children in all domains of the PedsQL MFS, with the exception of the cognitive fatigue domain. Specifically, 62.5% of the children age 2–7 were reported to be severely fatigued by their parents, compared to only 2.7% of healthy age controls (*p* < 0.005).

Similarly, the parents of children 8–18 years of age with PID reported significantly more fatigue for their children on all subscales compared to the healthy peer group (38.9% versus 2.9%, respectively; *p* < 0.005).

### The Relationship Between Self-Reported General Fatigue by Children with PID and Both HRQoL, and School Absences

We found that increased fatigue was associated with lower HRQoL (Table 3). Specifically, each 1-point reduction in the general PedsQL MFS score was associated with a mean reduction in that child’s total HRQoL score of 1.1 points (95% CI: 0.9–1.4 points). In addition, children with lower general fatigue scores (indicating more fatigue) had a similar association with respect to all four subscales on the PedsQL GCS (Table 3).

**Table 3:** Association between general fatigue and HRQoL and school absences among the pediatric patients with PID based on self-reported data

		Adjusted model^a^		
	Mean ± SD	B	95% CI	*p* value
PedsQL GCS (range 0–100)^b^ *8–18 years of age*				
Total HRQoL (*n* = 52)	74.5 ± 13.9	1.12	0.87; 1.36	**<0.001**
Physical functioning (*n* = 52)	76.3 ± 17.0	0.80	0.56; 1.04	**<0.001**
Emotional functioning (*n* = 51)	72.1 ± 19.2	0.47	0.21; 0.73	**0.001**
Social functioning (*n* = 52)	82.3 ± 15.2	0.74	0.43; 1.05	**<0.001**
School functioning (*n* = 52)	66.2 ± 18.4	0.85	0.65; 1.04	**<0.001**
School absence—*8–18 years of age*				
School absence in the past 6 months, % (*n* = 41)	11.0 ± 15.9	−0.47	−0.86; −0.08	**0.02**
School absence in the past 2 weeks, % (*n* = 40)	17.0 ± 23.8	−0.42	−0.68; −0.16	**0.002**

Significant results *p* < .05 are presented in bold

Abbreviations: *HRQoL*, health-related quality of life; *PedsQL GCS*: PedsQL generic core scales

^a^Adjusted for age and sex

^b^PedsQL is scored on a range of 0 to 100, with lower scores indicating reduced HRQoL

Moreover, children with lower general fatigue scores (indicating more fatigue) had a higher percentage of school absences in both the previous 2 weeks and the previous 6 months (reference point: completion of questionnaires) compared to children with higher general fatigue scores (indicating less fatigue).

### The Relationship Between Parent-Proxy–Reported General Fatigue and HRQoL

Table 4 summarizes the association between parent-proxy–reported fatigue and HRQoL for the children with PID, with the data presented separately for the children 2–7 years of age and the children 8–18 years of age. For both age groups, lower general fatigue scores were found with respect to total HRQoL and all four PedsQL GCS subscales (Table 4).

**Table 4:** Association between general fatigue and HRQoL among pediatric patients with PID based on parent-proxy–reported data

		Adjusted model^a^		
	Mean ± SD	B	95% CI	*p* value
PedsQL GCS (range: 0–100)^b^—*2–7 years*				
Total HRQoL (*n* = 24)	63.4 ± 16.5	0.79	0.58; 1.36	**<0.001**
Physical functioning (*n* = 23)	60.1 ± 23.6	0.78	0.55; 1.0	**<0.001**
Emotional functioning (*n* = 24)	59.8 ± 17.1	0.63	0.11; 1.14	**0.020**
Social functioning (*n* = 24)	75.7 ± 19.2	0.53	0.06; 1.0	**0.029**
School functioning (*n* = 23)	58.5 ± 19.6	0.84	0.15; 1.0	**0.011**
PedsQL GCS (range: 0–100)^b^—*8–18 years*				
Total HRQoL (n = 54)	69.1 ± 17.6	1.15	0.95; 1.36	**<0.001**
Physical functioning (*n* = 54)	69.1 ± 21.7	0.82	0.61; 1.03	**<0.001**
Emotional functioning (*n* = 54)	68.2 ± 22.6	0.62	0.38; 0.86	**<0.001**
Social functioning (*n* = 54)	74.7 ± 20.4	0.82	0.58; 1.06	**<0.001**
School functioning (*n* = 53)	64.0 ± 22.1	0.77	0.55; 0.99	**<0.001**

Significant results *p* < .05 are presented in bold

Abbreviations: *HRQoL*, health-related quality of life; *PedsQL GCS*: PedsQL generic core scales

^a^Adjusted for age and sex

^b^PedsQL is scored on a range of 0 to 100, with lower scores indicating reduced HRQoL

**Table 5:** Association between general fatigue and disease activity and comorbidity in pediatric patients with PID

	Adjusted model^a^			
	Mean ± SD	B	95% CI	*p* value
Disease activity^b^				
*Self-reported 8–18 years (n = 52)*	1.27 ± 0.44	−7.92	−20.61; 4.77	0.215
*Parent-proxy–reported 2–7 years (n = 24)*	1.33 ± 0.48	−6.5	−26.59;14.48	0.545
*Parent-proxy–reported 8–18 years (n = 54)*	1.24 ± 0.43	−10.04	−25.52; 5.46	0.199
Comorbidity				
*Self-reported 8–18 years (n = 52)*	0.27 ± 0.45	−3.68	−16.31; 8.95	0.561
*Parent-proxy–reported 2–7 years (n = 24)*	0.08 ± 0.28	−16.75	−53.52; 20.03	0.354
*Parent-proxy–reported 8–18 years (n = 54)*	0.26 ± 0.44	−0.57	−15.76; 14.61	0.940

^a^Adjusted for age and sex

^b^Based on the disease activity score, ranging from 1 to 3 for low, moderate, and high (see Methods for details)

### The Association Between Fatigue and Disease Activity and Comorbidity Among Pediatric Patients with PID

Lastly, we examined whether fatigue was associated with disease activity and/or comorbidity in children with PID. Among the 79 patients for whom disease activity was reported, 72.2%, 26.6%, and 1.3% had low, moderate, and high levels of disease activity, respectively (Table 1). Our multivariable analysis revealed that disease activity was not significantly associated with self-reported fatigue, parent-reported fatigue for the children 2–7 years of age, or parent-reported fatigue for the children 8–18 years of age (Table 5).

In addition, a subgroup analysis of the 16 patients with one or more comorbidities and the 63 patients who did not present with a comorbid disease revealed no significant association between the presence of comorbidity and self-reported fatigue or parent-reported fatigue in either age group (Table 5). Nevertheless, it is important to note that the study was not powered to detect such associations with comorbidity; thus, these findings are descriptive at best.

## Discussion

In a cross-sectional study in children with different types of primary immunodeficiency, nearly 20% of participants reported experiencing severe fatigue. Moreover, this percentage was considerably higher (nearly 63%) when using parent-proxy–reported data. Given that both of these percentages are significantly higher than those reported for healthy peers, it is reasonable to consider fatigue a clinically relevant issue among children with PID. Furthermore, we found that fatigue among children with PID was associated with both a significant increase in school absences and a significant decrease in health-related quality of life. Finally, we found no significant association between severe fatigue and either disease activity or the presence of comorbidity.

The results obtained in the pediatric patient population we studied are consistent with previous studies regarding fatigue in children and adults with PID [4, 9, 24], as well as regarding the effect of fatigue on HRQoL [29]. Importantly, some patients reported disabilities associated with fatigue, even when their disease was well-managed. These fatigue-related difficulties reflect the outcome of previous studies, which found that fatigue, an increased number of school absences, and lower HRQoL are related to chronic diseases in general and are therefore not necessarily specific to PID [30–32]. These findings provide new insights regarding the treatment of severe fatigue in children with chronic disease, in which a transdiagnostic approach to fatigue focusing on the individual patient’s needs is recommended over a specific disease-based approach, given that the disease itself is managed optimally [33].

Although the study was not powered to compare levels of severe fatigue between the different categories of immunodeficiencies, we observed that severe fatigue was reported on self- and parent-proxy reports in CVID, CID, and SIgA-deficient patients alike, but was not reported in XLA patients. Thus, although observed more often in patients with humoral immunodeficiencies, severe fatigue was not strictly related to the type and severity of immune dysfunction in our cohort. Since all XLA, CVID, and CID patients received immunoglobulin replacement therapy, versus none of the SIgA deficient patients, we conclude that persistent severe fatigue was not necessarily related to the use of immunoglobulin replacement therapy.

We speculate that the severe fatigue reported in the CVID and CID patients may be related in part to immune dysregulation observed frequently in these patients. This would be compatible with the observation that none of the XLA patients studied reported fatigue; on the other hand, this would not account for the observation that a significant percentage of the SIgA-deficient patients reported severe fatigue. Future studies could explore a possible relation between the presence or absence of immune dysregulation, its treatment (e.g., PID patients on subcutaneous versus intravenous immunoglobulin replacement) and treatment response, and the presence of severe fatigue in immunodeficiency patients.

Importantly, we found differences between the self-reported data and the parent-proxy–reported data. This finding is consistent with other studies that evaluated parent-child agreement with respect to the child’s HRQoL, showing that parents tend to underestimate their child’s HRQoL and functioning [34]. This difference in reporting outcome may be due to differences between parent and child with respect to reasoning and/or the response reactions [35]. Moreover, studies have shown that parental functioning (including mental functioning) and the parent’s quality of life can affect proxy reporting [17]. Thus, the perspectives of both the child and the parents should be considered, and parents should be actively involved in the treatment. Because disagreement between parent and child can potentially reduce efficacy with respect to treating the child’s fatigue and fatigue-related effects on functioning, this is an important point that warrants further study.

Psychosocial factors such as coping and cognitive health beliefs and biological factors are equally important, as conceptualized in the so-called biopsychosocial model [36, 37]. Thus, measuring disease activity, as well as other biological factors such as physical functioning and sleep as a fatigue-associated component, is an essential element in the biopsychosocial model. We therefore determined disease activity and the presence of comorbid disease in each patient and then analyzed their associations with fatigue.

Our study included a relatively large number of pediatric patients with PID, a rare condition; moreover, the high rate of participation (87%) reduces the likelihood of participation bias. However, the findings of severe fatigue between the different categories of immunodeficiencies (e.g., XLA patients) are descriptive and exploratory at best due to the modest size of our study population. In addition, we assessed fatigue in children ranging from 2 to 18 years of age, finding that fatigue is prevalent even in young children, thus adding to previous studies, the majority of which focused on studying fatigue in adolescents. The relatively high prevalence of fatigue in younger children suggests that the focus should shift from treating fatigue to preventing this debilitating symptom.

Our study has several caveats that warrant explanation. First, our study presented a nearly homogenous ethnic group (Caucasian). Therefore, these results cannot be generalized to other ethnic groups. Second, disease activity was relatively low among our pediatric patients with PID; 72.2% and 26.6% of our patients had “low” and “moderate” disease activity, respectively, based on hospitalization data and the medications used in a 3-month window surrounding the time in which the questionnaires were completed. Thus, our results may not necessarily be applicable to patients with markedly higher levels of disease activity. In addition, the fact that disease activity may change over time was not addressed in this cross-sectional study. Longitudinal follow-up with a regularly scheduled reassessment of the same subjects is thus recommended in future studies. Third, we were unable to control whether the parents helped their child complete the self-report questionnaires, as the questionnaires were completed at their homes. However, the parents were explicitly instructed not to help their child, and the relatively large discrepancies between the self-reported and parent-proxy–reported data suggest that the parents and children likely completed the questionnaires independently.

In conclusion, this study shows that fatigue is both prevalent and relevant in pediatric patients with PID. Specifically, we found that severe fatigue was associated with increased school absences and negatively affected the child’s quality of life, independent of disease activity and the presence of a comorbid disease. We therefore suggest that addressing fatigue in children and adolescents with PID, irrespective of the subtype of PID, is highly relevant from a clinical perspective. Our results also suggest that fatigue may be a promising target for therapeutic interventions, thereby improving functioning, increasing school participation, and improving quality of life in children with PID.

## Abbreviations

CIS-8: Checklist individual strength, 8-item subscale
CID: Combined immunodeficiency
CVID: Common variable immunodeficiency
HRQoL: Health-related quality of life
PedsQL GCS: Pediatric quality of life inventory generic core scales 4.0
PedsQL MFS: Pediatric quality of life inventory multidimensional fatigue scale 3.0
PID: Primary immunodeficiency
SIgAD: Selective immunoglobulin A deficiency
VAS: Visual analog scale
XLA: X-linked agammaglobulinemia
